# Deep learning using nasal endoscopy and T2-weighted MRI for prediction of sinonasal inverted papilloma-associated squamous cell carcinoma: an exploratory study

**DOI:** 10.1186/s41747-025-00610-0

**Published:** 2025-07-21

**Authors:** Jiliang Ren, Zhe Ren, Duo Zhang, Ying Yuan, Meng Qi

**Affiliations:** 1 Department of Radiology, Shanghai Ninth People’s Hospital, Shanghai Jiao Tong University School of Medicine, Shanghai, China; 2 Department of Otolaryngology–HNS, Eye & ENT Hospital, Fudan University, Shanghai, China; 3 Department of Radiology, Eye & ENT Hospital, Fudan University, Shanghai, China

**Keywords:** Deep learning, Endoscopy, Magnetic resonance imaging, Sinonasal inverted papilloma, Squamous cell carcinoma

## Abstract

**Background:**

Detecting malignant transformation of sinonasal inverted papilloma (SIP) into squamous cell carcinoma (SIP-SCC) before surgery is a clinical need. We aimed to explore the value of deep learning (DL) that leverages nasal endoscopy and T2-weighted magnetic resonance imaging (T2W-MRI) for automated tumor segmentation and differentiation between SIP and SIP-SCC.

**Methods:**

We conducted a retrospective analysis of 174 patients diagnosed with SIPs, who were divided into a training cohort (*n* = 121) and a testing cohort (*n* = 53). Three DL architectures were utilized to train automated segmentation models for endoscopic and T2W-MRI images. DL scores predicting SIP-SCC were generated using DenseNet121 from both modalities and combined to create a dual-modality DL nomogram. The diagnostic performance of the DL models was assessed alongside two radiologists, evaluated through the area under the receiver operating characteristic curve (AUROC), with comparisons made using the Delong method.

**Results:**

In the testing cohort, the FCN_ResNet101 and VNet exhibited superior performance in automated segmentation, achieving mean dice similarity coefficients of 0.95 ± 0.03 for endoscopy and 0.93 ± 0.02 for T2W-MRI, respectively. The dual-modality DL nomogram based on automated segmentation demonstrated the highest predictive performance for SIP-SCC (AUROC 0.865), outperforming the radiology resident (AUROC 0.672, *p* = 0.071) and the attending radiologist (AUROC 0.707, *p* = 0.066), with a trend toward significance. Notably, both radiologists improved their diagnostic performance with the assistance of the DL nomogram (AUROCs 0.734 and 0.834).

**Conclusion:**

The DL framework integrating endoscopy and T2W-MRI offers a fully automated predictive tool for SIP-SCC.

**Relevance statement:**

The integration of endoscopy and T2W-MRI within a well-established DL framework enables fully automated prediction of SIP-SSC, potentially improving decision-making for patients with suspicious SIP.

**Key Points:**

- Detecting the transformation of SIP into SIP-SCC before surgery is both critical and challenging.
- Endoscopy and T2W-MRI were integrated using DL for predicting SIP-SCC.
- The dual-modality DL nomogram outperformed two radiologists.
- The nomogram may improve decision-making for patients with suspicious SIP.

**Graphical Abstract:**

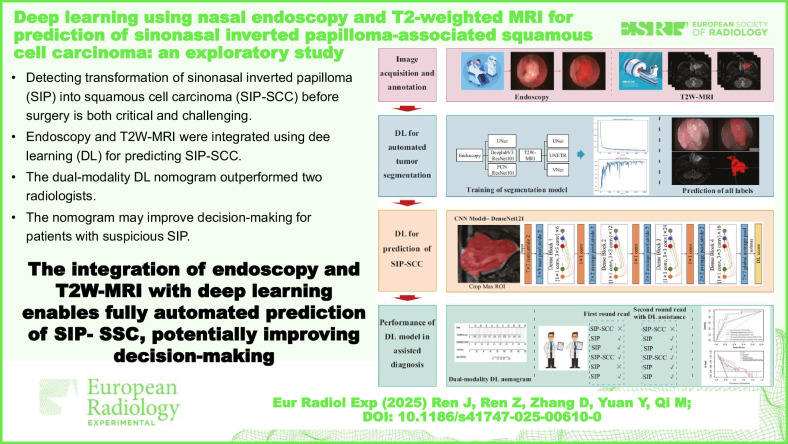

## Background

Sinonasal inverted papilloma (SIP) is the most common benign neoplasm found in the nasal cavity and paranasal sinuses [1], comprising up to 7% of all primary sinonasal tumors [2]. A significant concern with SIP is its potential for malignant transformation into squamous cell carcinoma (SIP-SCC) [3]. Compared with SIP, SIP-SCC requires different treatment approaches and has a poorer prognosis [4]. The management of SIP-SCC typically involves more extensive surgical resection, often accompanied by postoperative adjuvant radiotherapy or chemoradiotherapy [3, 5–7]. The high local failure rate associated with SIP-SCC can, in part, be attributed to misdiagnosis prior to surgery, which leads to inappropriate treatment for affected patients [6]. Consequently, early identification of SIP-SCC in the preoperative phase is crucial. Although preoperative punch biopsy is currently the standard diagnostic method, it has significant drawbacks, including low accuracy, the potential for sampling errors, and its invasive nature [8, 9].

Two commonly used diagnostic modalities for SIP are white light nasal endoscopy and magnetic resonance imaging (MRI) [9–11]. Nasal endoscopy offers a direct, microscopic view of the tumor’s surface features [8], while MRI provides a comprehensive assessment of the tumor’s overall and internal structure [12]. Together, these modalities deliver complementary, multidimensional information about sinonasal tumors. However, human visual assessment is often limited by personal experience, resulting in subjectivity and the potential to overlook critical information in medical images.

Deep learning (DL) has shown great promise in objectively and comprehensively extracting detailed information from medical images, particularly in the analysis of nasal lesions [13–18]. Recent studies have successfully employed DL algorithms for the automated detection and classification of nasal polyps and SIP in nasal endoscopic images [2]. Additionally, one study demonstrated the ability to differentiate between SIP and SIP-SCC using a three-dimensional (3D) convolutional neural network based on MRI data, achieving an area under the receiver operating characteristic curve (AUROC) of 0.80 in the test set [19]. However, to date, no studies have integrated MRI and nasal endoscopic images to accurately differentiate between SIP and SIP-SCC using DL algorithms. Furthermore, automated lesion segmentation in medical images is essential for precise diagnosis, yet studies successfully implementing automated sinonasal tumor segmentation across both endoscopy and MRI remain scarce.

This study aimed to explore the utility of DL relying on nasal endoscopy and T2-weighted (T2W) MRI for the automated segmentation of tumors and the discrimination between SIP and SIP-SCC.

## Methods

### Patients

**Fig. 1:**
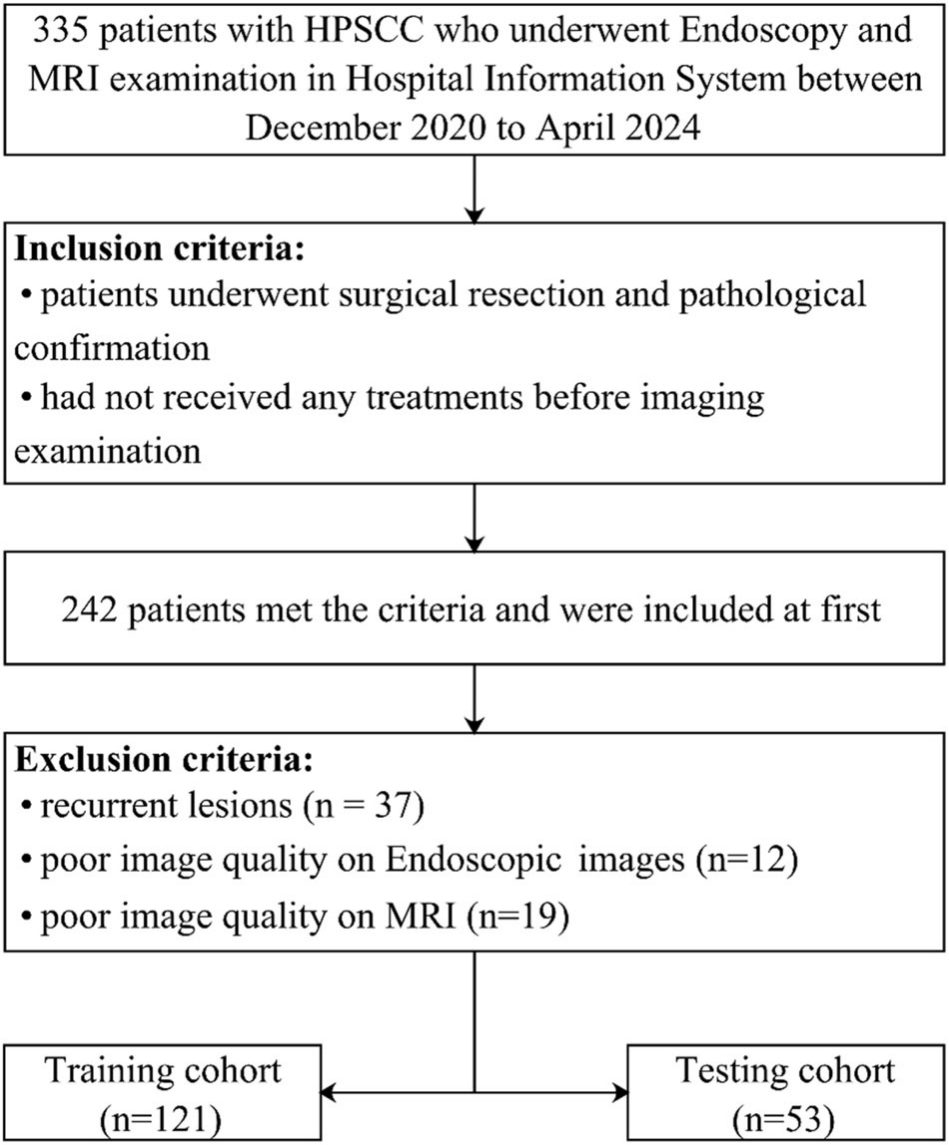
Flowchart of the patient selection process

This retrospective study was approved by the Ethics Review Board of Shanghai Eye & ENT Hospital of Fudan University, which waived the requirement for informed consent. We conducted a comprehensive search of the hospital information system to identify patients with SIPs from December 2020 to April 2024. Inclusion criteria were: (1) patients who underwent surgical resection with pathological confirmation, and (2) patients who had not received any treatment prior to imaging examination. Exclusion criteria were: (1) recurrent lesions; (2) endoscopic images with poor quality, manifested as excessive mucus, bleeding, or nasal hairs obscuring the lesion, or poor lighting conditions that impeded accurate lesion assessment; and (3) T2W images with poor quality, such as severe artifacts (motion artifacts or metal artifacts) or a low signal-to-noise ratio. Representative endoscopic or MRI images with poor image quality are presented in Supplementary Fig. S1. All patients were enrolled in the study and randomly assigned to either a training cohort or a testing cohort in a 7:3 ratio. A flowchart detailing patient selection is presented in Fig. 1.

### Image acquisition and annotation

MRI was performed using two 3.0-T Siemens MRI systems (Magnetom Verio or Prisma; Siemens Medical) equipped with a 12-channel and a 64-channel head and neck coil. The axial fat-suppressed T2W images were analyzed. The acquisition parameters included: repetition time 4,000–4,500 ms; echo time 99–120 ms; matrix size 640 × 592, field of view 220 × 220 mm^2^, slice thickness 6 mm, and interslice gap 0.6 mm. For annotation of T2W images, two radiologists, one with 7 years and the other with 9 years of experience in head and neck imaging diagnosis, collaboratively outlined the tumor contours layer by layer to define the region of interest (ROI) using ITK-SNAP software (www.itk-snap.org). During the annotation process, any discrepancies, such as the determination of tumor boundaries or the exclusion of non-tumor tissues like sinusitis or normal structures, were resolved by a senior radiologist who had over 20 years of experience.

Endoscopic examinations were performed using a STORZ endoscope (Xenon Nova 175, KARL STORZ). Images were captured in JPG format with three color channels, with dimensions ranging from 600 to 1,000 pixels. An otolaryngologist with 7 years of experience chose the most representative endoscopic images for each patient from four in the reporting system. The selection criteria were clear: maximum tumor exposure to show macroscopic features and minimal obscuring elements like mucus or nasal hair. This doctor then outlined the tumor contours using Labelme software (labelme.csail.mit.edu). Next, an otolaryngologist with 9 years of experience reviewed, confirmed the selected images, and refined the annotations. Moreover, a 5-year-experienced otolaryngologist randomly picked images from 40 patients to evaluate the inter-rater reliability in the image-selection process.

### DL for automated segmentation

Before training the segmentation model, T2W-MRI images underwent preprocessing. First, the anatomical orientations of MRI images and corresponding labels were normalized to the RAS (right, anterior, and superior) axis codes. The voxel sizes were then resampled to a uniform dimension of 1 mm × 1 mm × 1 mm, using bilinear interpolation for MRI images and nearest neighbor interpolation for labels. Finally, intensity values were standardized to a range of 0 to 1,600 through linear transformation, with an optional clipping step to address outliers.

For endoscopic image segmentation, we applied the UNet, DeepLabV3_ResNet101, and FCN_ResNet101 models, while the UNet, UNETR, and VNet models were used for MRI image segmentation. During training, we selectively cropped sub-volumes from both endoscopic and MRI images along with their labels to balance positive and negative samples. To enhance the diversity of the training dataset, we implemented online data augmentation strategies, such as spatial modifications and random cropping, ensuring varied image sets for each training iteration. For hyperparameter tuning, we set a learning rate of 1e-3 and employed the Adam optimizer with a batch size of 4. To mitigate overfitting, early stopping was enforced after 32 epochs. The DiceCE Loss, a hybrid loss function combining Dice and cross-entropy [20], was utilized. Losses were employed to manage class imbalance effectively and enhance segmentation precision. The model’s effectiveness in automatically delineating ROIs was evaluated using the Dice similarity coefficient (DSC) and mean intersection over union (mIoU) metrics.

### DL for prediction of SIP-SCC

Within the transfer learning framework, the DenseNet121 convolutional neural network was utilized, initialized with weights from ImageNet. Classification models were trained using two sets of ROIs: manual annotations and automated segmentations. For MRI images, only the largest tumor slice was analyzed. To minimize background noise and concentrate on critical areas, the minimum bounding rectangle surrounding the ROI in both MRI and endoscopic images was preserved and expanded outward by 5 pixels, ensuring inclusion of the area surrounding the tumor. During preprocessing, images underwent Z-score normalization to eliminate intensity variations. For the training cohort, data augmentation techniques—such as random cropping, horizontal flipping, and vertical flipping—were implemented to enhance data diversity and improve the model’s generalization ability. To reduce the impact of imbalanced sample classification on model building, we oversampled the minority SIP-SCC class using the Synthetic Minority Over-sampling Technique (SMOTE). This involved generating synthetic samples to boost their presence in the training cohort. After training, the DL score, representing the predictive value, was generated following the sequential activation of convolutional and pooling layers.

The DL scores from endoscopy and T2W MRI, based on automated segmentation, were integrated using a logistic regression model to create a dual-modality nomogram. To improve transparency in the model’s decision-making process and enhance interpretability, the gradient-weighted class activation mapping (Grad-CAM) method was combined with a localized attention map to identify crucial regions within the classified target images. The development of DL models was conducted using Python (version 3.7.12, www.python.org) and the OnekeyAI platform (version 3.1.8, www.medai.icu or github.com/OnekeyAI-Platform/onekey).

### Performance of the DL model in assisted diagnosis

**Fig. 2:**
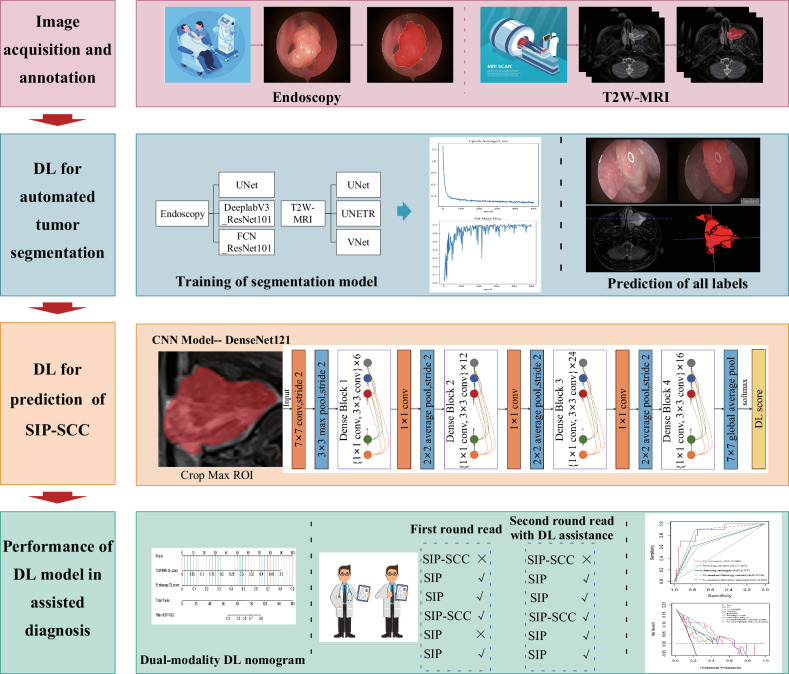
Overall pipeline of this study. DL, Deep learning

All conventional MRI images of the patients were independently reviewed by a radiology resident and an attending radiologist, both blinded to the histopathological results. The tumor categorization (SIP *versus* SIP-SCC) was determined based on morphological features. After one week, both radiologists reassessed the MRI images for a second diagnosis, this time considering the predictive results generated by the DL nomogram as a reference. The data analysis workflow is illustrated in Fig. 2.

### Statistical analysis

All statistical analyses were conducted using R software (version 3.5.2; www.r-project.org). Comparisons of clinical characteristics between the two groups were assessed using the Mann–Whitney *U*-test or the chi-squared test, as appropriate. The kappa value was calculated to assess the consistency in the selection of endoscopic images between two otolaryngologists. Receiver operating characteristic (ROC) curves evaluated the predictive performance of the DL models and the radiologists, with areas under the curves (AUROCs) compared using the DeLong test. The Hosmer–Lemeshow test assessed the calibration of the DL nomogram. Decision curve analysis quantified the net benefit from utilizing the DL nomogram and the two radiologists at varying threshold probabilities. A *p*-value of less than 0.05 indicated statistical significance.

## Results

### Patients’ characteristics

The study involved a training cohort (*n* = 121) and a testing cohort (*n* = 53). The training cohort comprised 97 SIPs and 24 SIP-SCCs, while the testing cohort consisted of 43 SIPs and 10 SIP-SCCs. No statistically significant differences were found in age, gender, or pathological classification between the cohorts (all *p* ≥ 0.230). Table 1 summarizes the baseline characteristics of all participants.

**Table 1:** Clinical characteristics of patients

Characteristic	All patients (*n* = 174)	Training cohort (*n* = 121)	Validation cohort (*n* = 53)	*p*-value
Sex				0.230
Female	52 (29.9%)	40 (33.1%)	12 (22.6%)	
Male	122 (70.1%)	81 (66.9%)	41 (77.4%)	
Age (years)	56 (42, 67)	58 (49, 65)	57 (48, 62)	0.449
Pathological type				0.952
SIP	140 (80.5%)	97 (80.2%)	43 (81.1%)	
SIP-SCC	34 (19.5%)	24 (19.8%)	10 (18.9%)	

Data are expressed as median (interquartile range) or number (percentage)

*SIP* Sinonasal inverted papilloma, *SCC* Squamous cell carcinoma

### Performance of DL for automated segmentation

For automated segmentation of endoscopic images, the FCN_ResNet101 model demonstrated the highest performance, achieving a DSC of 0.94 ± 0.05 and an mIoU of 0.89 ± 0.08 in the training cohort, and 0.95 ± 0.03 and 0.90 ± 0.06, respectively, in the testing cohort. In T2W-MRI image segmentation, the VNet model outperformed others, with DSC and mIoU values of 0.91 ± 0.10 and 0.86 ± 0.10 in the training cohort, and 0.93 ± 0.02 and 0.88 ± 0.03 in the testing cohort. Performance metrics for DL models in automated segmentation are detailed in Table 2.

**Table 2:** Performance of DL models for automated tumor segmentation

Cohorts	Endoscopy			T2W-MRI		
	Model	DSC	mIoU	Model	DSC	mIoU
Training cohort	UNet	0.70 ± 0.20	0.57 ± 0.21	UNet	0.87 ± 0.09	0.81 ± 0.09
Training cohort	DeeplabV3_ResNet101	0.94 ± 0.04	0.89 ± 0.07	UNETR	0.82 ± 0.11	0.75 ± 0.10
Training cohort	FCN_ResNet101	0.94 ± 0.05	0.89 ± 0.08	VNet	0.91 ± 0.10	0.86 ± 0.10
Testing cohort	UNet	0.68 ± 0.21	0.55 ± 0.22	UNet	0.89 ± 0.04	0.82 ± 0.05
Testing cohort	DeeplabV3_ResNet101	0.91 ± 0.04	0.86 ± 0.07	UNETR	0.86 ± 0.05	0.78 ± 0.06
Testing cohort	FCN_ResNet101	0.95 ± 0.03	0.90 ± 0.06	VNet	0.93 ± 0.02	0.88 ± 0.03

Data are presented as numbers (%) or mean ± standard deviation

*DSC* Dice similarity coefficient, *mIoU* Mean intersection over union

### Performance of DL for predicting SIP-SCC

**Fig. 3:**
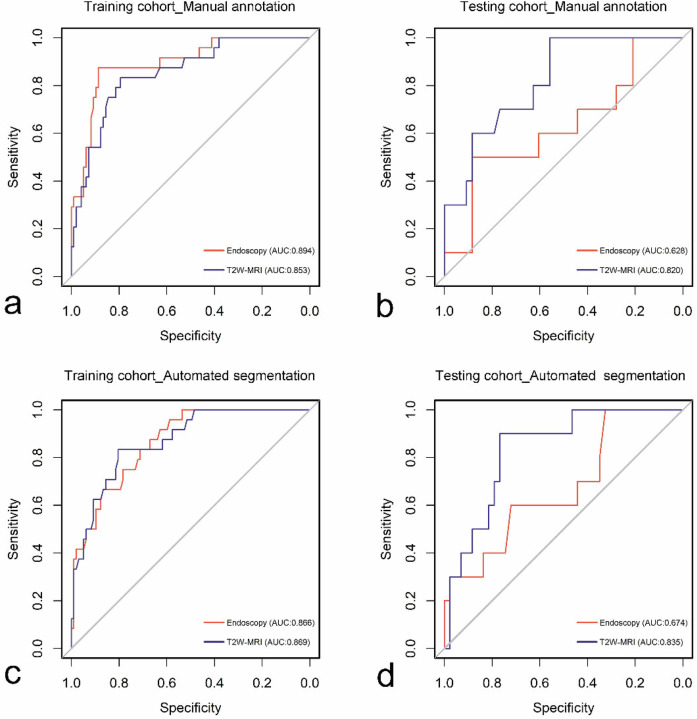
ROC curves for the endoscopy and T2W MRI models based on manual annotation (**a**, **b**) and automated segmentation (**c**, **d**) in the training (**a**, **c**) and testing (**b**, **d**) cohorts, respectively

Overall, the DL models based on automated segmentation demonstrated comparable predictive performance to those utilizing manual annotation. Notably, in the testing cohort, the T2W-MRI model outperformed the endoscopy model across both segmentation methods. With manual annotation, the endoscopy and T2W-MRI models yielded AUROCs of 0.894 and 0.853 in the training cohort, respectively, and 0.628 and 0.820 in the testing cohort. Using automated segmentation, the AUROCs for the endoscopy and T2W-MRI models were 0.866 and 0.869 in the training cohort, and 0.674 and 0.835 in the testing cohort. There were no significant differences in AUROCs between the DL models based on manual annotation and those based on automated segmentation in the testing cohort (*p* = 0.369 and 0.841). When evaluating the consistency in endoscopic image selection between the two otolaryngologists, there was an almost perfect agreement with a κ value of 0.886. The performance data for the endoscopy and T2W-MRI models is summarized in Table 3, with corresponding curves illustrated in Fig. 3.

**Table 3:** Predictive performance of DL models based on DenseNet121

Cohorts	Segmentation	Modality	AUROC	Accuracy	Sensitivity	Specificity	PPV	NPV
Training cohort	Manual annotation	Endoscopy	0.894 (0.839–0.949)	0.884 (0.827–0.941)	0.875 (0.743–1.000)	0.887 (0.823–0.950)	0.656 (0.492–0.821)	0.966 (0.929–1.000)
	Manual annotation	T2W-MRI	0.853 (0.790–0.916)	0.802 (0.731–0.873)	0.833 (0.684–0.982)	0.794 (0.713–0.874)	0.500 (0.345–0.655)	0.951 (0.903–0.998)
	Automated segmentation	Endoscopy	0.866 (0.805–0.926)	0.661 (0.577–0.745)	0.958 (0.878–1.000)	0.588 (0.490–0.686)	0.365 (0.246–0.484)	0.983 (0.949–1.000)
	Automated segmentation	T2W-MRI	0.869 (0.808–0.929)	0.810 (0.740–0.880)	0.833 (0.684–0.982)	0.804 (0.725–0.883)	0.513 (0.356–0.670)	0.951 (0.905–0.998)
Testing cohort	Manual annotation	Endoscopy	0.628 (0.498–0.758)	0.811 (0.706–0.917)	0.500 (0.190–0.810)	0.884 (0.788–0.980)	0.500 (0.190–0.810)	0.884 (0.788–0.980)
	Manual annotation	T2W-MRI	0.820 (0.716–0.923)	0.642 (0.512–0.771)	1.000 (1.000–1.000)	0.558 (0.410–0.707)	0.345 (0.172–0.518)	1.000 (1.000–1.000)
	Automated segmentation	Endoscopy	0.674 (0.548–0.801)	0.453 (0.319–0.587)	1.000 (1.000–1.000)	0.326 (0.186–0.466)	0.256 (0.119–0.393)	1.000 (1.000–1.000)
	Automated segmentation	T2W-MRI	0.835 (0.735–0.935)	0.792 (0.683–0.902)	0.900 (0.714–1.000)	0.767 (0.641–0.894)	0.474 (0.249–0.698)	0.971 (0.914–1.000)

Data in parentheses are 95% confidence intervals

*AUROC* Area under the receiver operating characteristic curve, *PPV* Positive predictive value, *NPV* Negative predictive value

### Performance of the DL model in assisted diagnosis

**Fig. 4:**
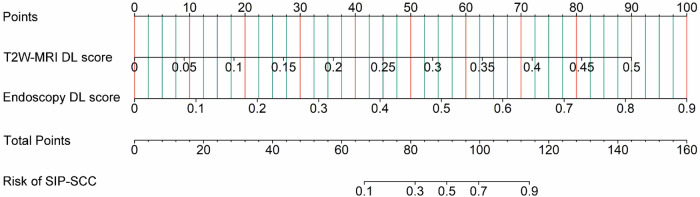
Dual-modality DL nomogram combining DL scores from the endoscopy and T2W MRI based on automated segmentation

**Fig. 5:**
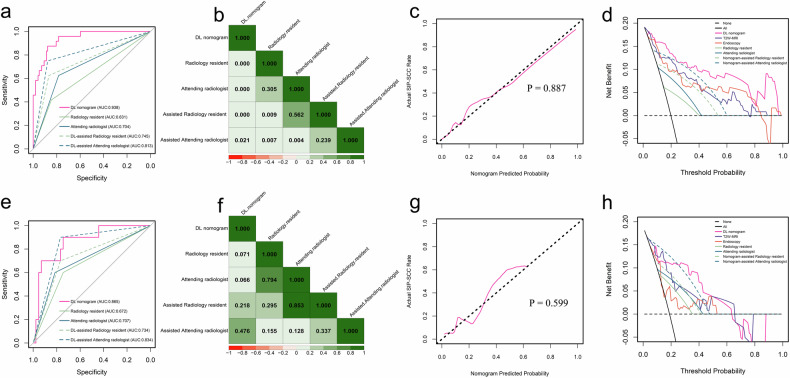
Performance of the dual-modality DL nomogram and two radiologists in the training (**a**–**d**) and testing (**e**–**h**) cohorts. **a**, **e** ROC curves. **b**, **f** Heatmaps based on the results of the DeLong tests. **c**, **g** Calibration curves of the DL nomogram. Calibration curves show excellent agreement between the nomogram-predicted and observed SIP-SCC probabilities. **d**, **h** Decision curves analysis (DCA) curves show that the nomogram had a higher overall net benefit than two single-modality modes and two radiologists across the majority of the range of reasonable threshold probabilities

**Fig. 6:**
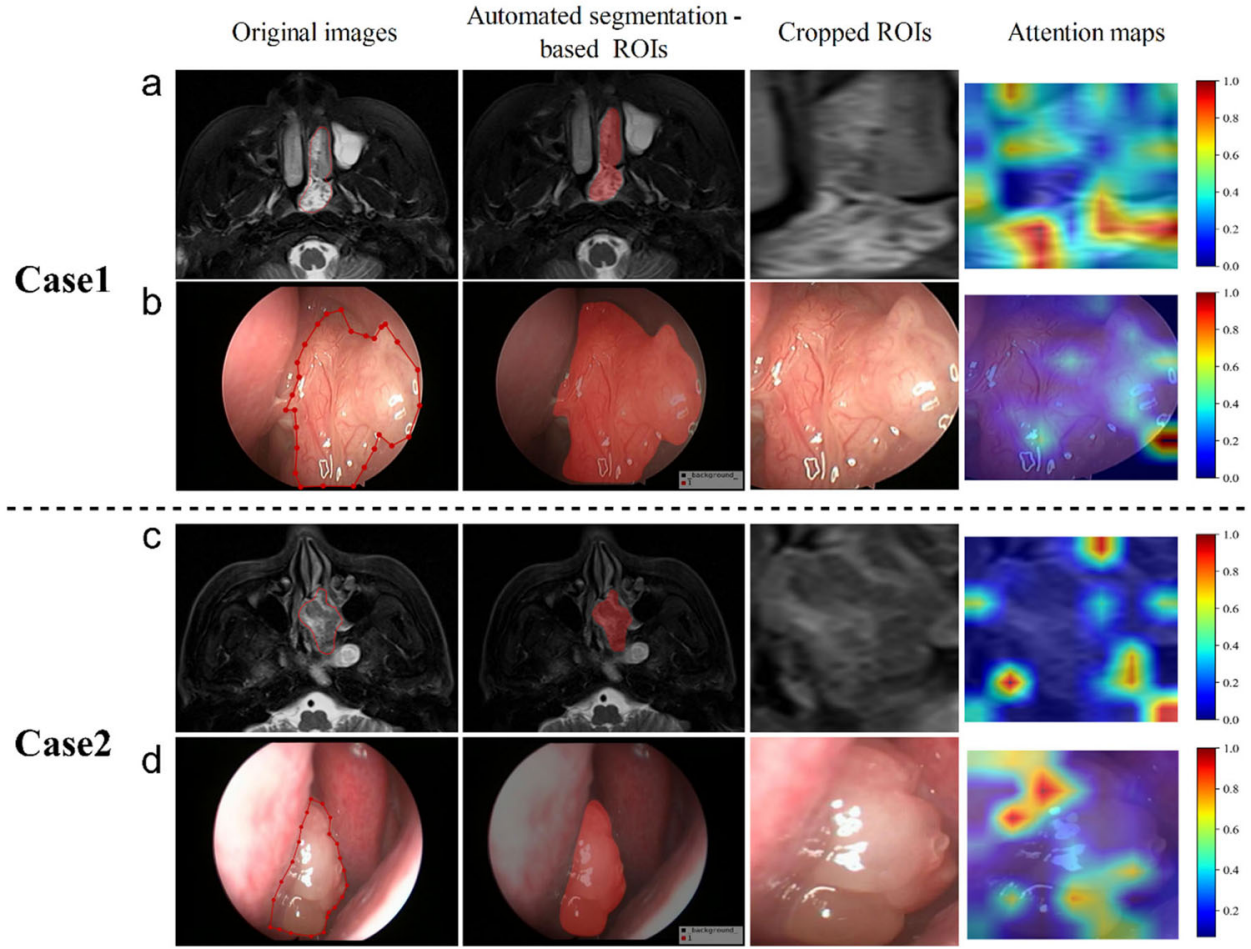
Representative cases of pathologically confirmed SIP (Case 1) and SIP-SCC (Case 2) were correctly diagnosed by the fully automated DL framework. Case 1 was correctly diagnosed by two doctors, but Case 2 was misdiagnosed by the radiology resident. The T2W magnetic resonance images (rows **a**, **c**) and endoscopic (rows **b**, **d**) images from the two patients are displayed. Each image sequence from left to right shows the original images, automated segmentation-based regions of interest (ROIs), cropped ROIs, and attention maps. In the attention maps displayed as heat maps, warmer colors indicate a higher contribution to the classification decision

The DL scores from the endoscopy and T2W-MRI models, based on automated segmentation, were combined to construct a dual-modality nomogram (Fig. 4). This nomogram demonstrated optimal prediction performance, with AUROCs of 0.938 and 0.865 in the training and testing cohorts, respectively. The radiology resident and attending radiologist had AUROCs of 0.631 and 0.704 in the training cohort, and 0.672 and 0.707 in the testing cohort. The differences in AUROC between the DL nomogram and the radiology resident and attending radiologist trended towards significance in the testing cohort (*p* = 0.071 and 0.066, respectively), while no significant differences were observed when both doctors used the DL nomogram (*p* = 0.218 and 0.476). With the DL nomogram’s assistance, both radiologists improved their diagnostic performance, achieving AUROCs of 0.745 and 0.813 in the training cohort, and 0.734 and 0.834 in the testing cohort. Notably, according to the Delong test, significant differences were observed in certain AUROCs when comparing radiologists working independently to those utilizing DL assistance. The performances of the DL nomogram and the two radiologists are detailed in Table 4. ROC curves for the DL nomogram and the two radiologists are presented in Fig. 5a, e, and the heatmaps based on the results of the Delong tests are displayed in Fig. 5b, f. The DL nomogram exhibited good calibration, yielding non-significant results in both cohorts (*p* = 0.887 and 0.599) as shown in Fig. 5c, g. Decision curve analysis indicated that the DL nomogram provided a higher overall net benefit than two single–modality modes and two radiologists across a range of reasonable threshold probabilities in both cohorts (Fig. 5d, h). Representative cases accurately diagnosed using the automated DL framework are illustrated in Fig. 6.

**Table 4:** Diagnostic performance of the dual-modality DL nomogram and two radiologists

Cohorts		AUROC	Accuracy	Sensitivity	Specificity	PPV	NPV
Training cohort	Dual-modality DL nomogram	0.938 (0.895–0.981)	0.876 (0.817–0.935)	0.875 (0.743–1.000)	0.876 (0.811–0.942)	0.636 (0.472–0.800)	0.966 (0.928–1.000)
	Radiology resident	0.631 (0.545–0.717)	0.760 (0.684–0.836)	0.417 (0.219–0.614)	0.845 (0.773–0.917)	0.400 (0.208–0.592)	0.854 (0.784–0.925)
	Attending radiologist	0.704 (0.623–0.786)	0.752 (0.675–0.829)	0.625 (0.431–0.819)	0.784 (0.702–0.865)	0.417 (0.256–0.578)	0.894 (0.829–0.960)
	Nomogram-assisted radiology resident	0.745 (0.668–0.823)	0.818 (0.749–0.887)	0.625 (0.431–0.819)	0.866 (0.798–0.934)	0.536 (0.351–0.720)	0.903 (0.843–0.963)
	Nomogram-assisted attending radiologist	0.813 (0.744–0.883)	0.851 (0.788–0.915)	0.750 (0.577–0.923)	0.876 (0.811–0.942)	0.600 (0.425–0.775)	0.934 (0.883–0.985)
Testing cohort	Dual-modality DL nomogram	0.865 (0.773–0.957)	0.774 (0.661–0.886)	0.900 (0.714–1.000)	0.744 (0.614–0.875)	0.450 (0.232–0.668)	0.970 (0.911–1.000)
	Radiology resident	0.672 (0.546–0.798)	0.717 (0.596–0.838)	0.600 (0.296–0.904)	0.744 (0.614–0.875)	0.353 (0.126–0.580)	0.889 (0.786–0.992)
	Attending radiologist	0.707 (0.584–0.830)	0.774 (0.661–0.886)	0.600 (0.296–0.904)	0.814 (0.698–0.930)	0.429 (0.169–0.688)	0.897 (0.802–0.993)
	Nomogram-assisted radiology resident	0.734 (0.615–0.853)	0.755 (0.639–0.871)	0.700 (0.416–0.984)	0.767 (0.641–0.894)	0.412 (0.178–0.646)	0.917 (0.826–1.000)
	Nomogram-assisted attending radiologist	0.834 (0.733–0.934)	0.792 (0.683–0.902)	0.900 (0.714–1.000)	0.767 (0.641–0.894)	0.474 (0.249–0.698)	0.971 (0.914–1.000)

Data in parentheses are 95% confidence intervals

*AUROC* Area under the receiver operating characteristic curve, *PPV* Positive predictive value, *NPV* Negative predictive value

## Discussion

In this research, we crafted a novel DL system that uses endoscopy and T2W-MRI to automatically predict SIP-SCC. The FCN_ResNet101 model excels at segmenting tumors in endoscopic images, and the VNet model performs well in T2W-MRI images. A dual-modality DL nomogram based on automated segmentation performed best in both training and testing cohorts, with AUROCs of 0.938 and 0.865, respectively. This nomogram can assist both novice and experienced radiologists in achieving more accurate diagnoses. As far as we know, this is the first study integrating endoscopic and MRI data for fully automatic SIP-SCC diagnosis.

In our study, we employed three distinct algorithms to train segmentation models specifically for two-dimensional endoscopic images and 3D T2W-MRI images. The FCN_ResNet101 model demonstrated the highest performance for two-dimensional endoscopic images, achieving a DSC value of 0.95 ± 0.03, while the VNet model excelled in 3D T2W images, with a DSC value of 0.93 ± 0.02 in the testing cohort. Both FCN_ResNet101 and VNet are fully convolutional networks recognized as state-of-the-art methods for medical image segmentation in recent years [21]. Their residual mechanisms effectively address the vanishing-gradient problem associated with deeper networks, enhancing feature propagation and learning [22, 23]. Furthermore, both models utilize skip connections and multi-scale feature fusion, facilitating the extraction and integration of features at various levels, thereby improving segmentation outcomes [24]. In addition, our DL models for predicting SIP-SCC via automated detection perform comparably to those developed using manual annotation. This underscores the potential of the trained FCN_ResNet101 and VNet models to automate the segmentation of suspicious SIPs in endoscopic and T2W-MRI images, enabling rapid and effective diagnosis.

In the classification task of predicting SIP-SCC, we achieved satisfactory performance using a pre-trained DenseNet121 neural network. This architecture is characterized by its denser connectivity compared to conventional convolutional networks and naturally incorporates identity mappings, deep supervision, and varied depths [25]. Extensive transfer learning with DenseNet architectures has demonstrated promising results across various image analysis tasks, including tongue cancer detection [26], nasal cavity mass classification through endoscopic images [27], lymph node metastasis prediction in breast cancer [28], and glioma subtype classification using MRI images [29]. Our study utilized both endoscopy and T2W-MRI for model construction. The results revealed that, regardless of using manual annotation or automated segmentation, the T2W-MRI model (AUROCs 0.820 and 0.835) consistently outperformed the endoscopy model (AUROCs 0.628 and 0.674) for predicting SIP-SCC in the testing cohort. This advantage is primarily due to the limited view of tumor surface features provided by nasal endoscopy, while MRI offers a comprehensive perspective on the tumor’s overall appearance and internal structure. Nonetheless, the nomogram that combined DL scores from both modalities achieved an enhanced performance with an AUROC of 0.865 in the testing cohort, indicating that the two modalities provide complementary diagnostic information for SIP-SCC. Furthermore, despite the lower efficiency of DL with endoscopy, this simple examination is more convenient for initial SIP-SCC screening.

Given that certain morphological MRI features strongly indicate SIP-SCC diagnosis, we compared the DL nomogram’s performance with visual interpretations by two radiologists [30]. In the testing cohort, the DL nomogram (AUROC 0.865) outperformed both the radiology resident (AUROC: 0.672) and the attending radiologist (AUROC 0.707), though the significance trend was not fully clear (*p* = 0.071 and 0.066, respectively). This might be due to the small sample size, which led to a wide AUROC confidence interval, obscuring the clear demonstration of the DL model’s superiority. Additionally, the DL nomogram improved the diagnostic performance of both radiologists (AUROCs 0.734 and 0.834). Decision curve analysis also showed that the DL nomogram provided a superior overall net benefit compared to the radiologists’ visual interpretations. These results suggest that the DL nomogram is a reliable approach for predicting SIP-SC. However, further research with larger sample sizes is needed to better clarify the performance gap between the DL nomogram and radiologists.

Our DL model holds great promise for clinical use. It can screen sinonasal-symptom patients, helping clinicians prioritize biopsies. While histology remains the gold standard and our model won’t replace it, it’s valuable when biopsies are tough, like in complex anatomy or high-risk patients. In addition, the DL algorithm can integrate into Picture Archiving and Communication Systems or endoscopy viewers. For the former option, it offers real-time SIP-SCC predictions during image review, saving time and enabling earlier detection. Endoscopy offers real-time lesion feedback and provides a heatmap overlaid on the images, highlighting the regions that contribute significantly to the model’s diagnosis for better biopsy guidance. However, implementation has hurdles. We must cooperate with software vendors for smooth integration and refine the tech for real-time use. The model’s resource consumption extends image-analysis time, so we will optimize the algorithm. Finally, training clinicians on the model is also essential.

However, our study was not without limitations. Firstly, due to strict inclusion criteria, the sample size was relatively small for a DL study. To tackle this issue, in the future, we will collaborate with more than four centers located in diverse regions. We’ll apply our models to their datasets using current procedures, set up a standardized image pre-processing pipeline to reduce scanner-related grayscale differences, and use data augmentation to boost generalizability. After anonymizing the dataset, we’ll upload it to a public repository to encourage more researchers to partake in validating and refining the model. Secondly, our analysis focused on common endoscopic and T2W images. Incorporating CT and clinical data, such as the HPV status, is expected to improve the predictive accuracy. Thirdly, in consideration of computational efficiency and simplicity, we used only one MRI slice and one endoscopic image per patient, yet this might have caused the loss of contextual information. Future work should use 3D MRI data and multiple endoscopic images. Lastly, we used only DenseNet121 for training; exploring advanced architectures like vision transformers may refine results.

In conclusion, we developed a DL framework that integrates endoscopy and T2WI-MRI for the automated segmentation of tumors and the prediction of malignant transformation in suspicious SIPs. Our proposed dual-modality nomogram may enhance decision-making for patients with these suspicious lesions.

## Supplementary information

ELECTRONIC SUPPLEMENTARY MATERIAL

## Data Availability

The data that support the findings of this study are not publicly available due to privacy or ethical restrictions, but are available on request from the corresponding author.

## Abbreviations

3D: Three-dimensional
AUROC: Area under the receiver operating characteristic curve
DL: Deep learning
DSC: Dice similarity coefficient
mIoU: Mean intersection over union
MRI: Magnetic resonance imaging
ROC: Receiver operating
ROI: Region of interest
SCC: Squamous cell carcinoma
SIP: Sinonasal inverted papilloma
T2W: T2-weighted

## References

[1] Trent MS, Goshtasbi K, Hui L et al (2022) A systematic review of definitive treatment for inverted papilloma attachment site and associations with recurrence. Otolaryngol Head Neck Surg 167:425–433. 10.1177/01945998211051975 34637363 10.1177/01945998211051975

[2] Girdler B, Moon H, Bae MR et al (2021) Feasibility of a deep learning-based algorithm for automated detection and classification of nasal polyps and inverted papillomas on nasal endoscopic images. Int Forum Allergy Rhinol 11:1637–1646. 10.1002/alr.22854 34148298 10.1002/alr.22854

[3] Lee JJ, Peterson AM, Embry TW et al (2021) Survival outcomes of De Novo vs inverted papilloma-associated sinonasal squamous cell carcinoma: a systematic review and meta-analysis. JAMA Otolaryngol Head Neck Surg 147:350–359. 10.1001/jamaoto.2020.5261 33507208 10.1001/jamaoto.2020.5261 PMC7844698

[4] Birkenbeuel JL, Pang JC, Lee A et al (2022) Long-term outcomes in sinonasal squamous cell carcinoma arising from inverted papilloma: systematic review. Head Neck 44:1014–1029. 10.1002/hed.26995 35141984 10.1002/hed.26995

[5] Contrera KJ, Woody NM, Rahman M, Sindwani R, Burkey BB (2020) Clinical management of emerging sinonasal malignancies. Head Neck 42:2202–2212. 10.1002/hed.26150 32212360 10.1002/hed.26150

[6] Quan H, Zhang H, Zou L, Yuan W, Wang S (2020) Comparison of outcomes between patients with de-novo sinonasal squamous cell carcinoma vs malignant transformations from inverted papillomas. Int Forum Allergy Rhinol 10:762–767. 10.1002/alr.22556 32216167 10.1002/alr.22556

[7] Kamel RH, Khaled A, Abdelfattah AF, Awad AG (2022) Surgical treatment of sinonasal inverted papilloma. Curr Opin Otolaryngol Head Neck Surg 30:26–32. 10.1097/moo.0000000000000781 34889848 10.1097/MOO.0000000000000781

[8] Han MW, Lee BJ, Jang YJ, Chung YS (2010) Clinical value of office-based endoscopic incisional biopsy in diagnosis of nasal cavity masses. Otolaryngol Head Neck Surg 143:341–347. 10.1016/j.otohns.2010.05.019 20723769 10.1016/j.otohns.2010.05.019

[9] Park MJ, Cho W, Kim JH et al (2023) Preoperative prediction of sinonasal inverted papilloma-associated squamous cell carcinoma (IP-SCC). Laryngoscope 133:2502–2510. 10.1002/lary.30583 36683553 10.1002/lary.30583

[10] Wang M, Hou L, Zhou B et al (2021) Risk factors of malignant transformation of sinonasal inverted papilloma. Lin Chuang Er Bi Yan Hou Tou Jing Wai Ke Za Zhi 35:627–632. 10.13201/j.issn.2096-7993.2021.07.011 34304493 10.13201/j.issn.2096-7993.2021.07.011 PMC10127900

[11] Lisan Q, Laccourreye O, Bonfils P (2016) Sinonasal inverted papilloma: from diagnosis to treatment. Eur Ann Otorhinolaryngol Head Neck Dis 133:337–341. 10.1016/j.anorl.2016.03.006 27053431 10.1016/j.anorl.2016.03.006

[12] Zhang D, Zhang J, Zhou J et al (2023) Predictive value of magnetic resonance imaging multi-parametric analysis for malignant transformation of sinonasal inverted papilloma: a comprehensive prediction model. Curr Med Imaging 19:596–604. 10.2174/1573405618666220928091936 36173080 10.2174/1573405618666220928091936

[13] Wang K, Ren Y, Ma L et al (2023) Deep learning-based prediction of treatment prognosis from nasal polyp histology slides. Int Forum Allergy Rhinol 13:886–898. 10.1002/alr.23083 36066094 10.1002/alr.23083

[14] Kwon KW, Park SH, Lee DH et al (2024) Deep learning algorithm for the automated detection and classification of nasal cavity mass in nasal endoscopic images. PLoS One 19:e0297536. 10.1371/journal.pone.0297536 38478548 10.1371/journal.pone.0297536 PMC10936791

[15] Chowdhury NI, Smith TL, Chandra RK, Turner JH (2019) Automated classification of osteomeatal complex inflammation on computed tomography using convolutional neural networks. Int Forum Allergy Rhinol 9:46–52. 10.1002/alr.22196 30098123 10.1002/alr.22196 PMC6318014

[16] Nakagawa J, Fujima N, Hirata K et al (2022) Utility of the deep learning technique for the diagnosis of orbital invasion on CT in patients with a nasal or sinonasal tumor. Cancer Imaging 22:52. 10.1186/s40644-022-00492-0 36138422 10.1186/s40644-022-00492-0 PMC9502604

[17] Lin M, Lin N, Yu S et al (2023) Automated prediction of early recurrence in advanced sinonasal squamous cell carcinoma with deep learning and multi-parametric MRI-based radiomics nomogram. Acad Radiol 30:2201–2211. 10.1016/j.acra.2022.11.013 36925335 10.1016/j.acra.2022.11.013

[18] Lin N, Shi Y, Ye M, Wang L, Sha Y (2024) Multiparametric MRI-based radiomics approach with deep transfer learning for preoperative prediction of Ki-67 status in sinonasal squamous cell carcinoma. Front Oncol 14:1305836. 10.3389/fonc.2024.1305836 38939344 10.3389/fonc.2024.1305836 PMC11208468

[19] Liu GS, Yang A, Kim D et al (2022) Deep learning classification of inverted papilloma malignant transformation using 3D convolutional neural networks and magnetic resonance imaging. Int Forum Allergy Rhinol 12:1025–1033. 10.1002/alr.22958 34989484 10.1002/alr.22958

[20] Jin X, Thomas MA, Dise J et al (2021) Robustness of deep learning segmentation of cardiac substructures in noncontrast computed tomography for breast cancer radiotherapy. Med Phys 48:7172–7188. 10.1002/mp.15237 34545583 10.1002/mp.15237

[21] Drozdzal M, Chartrand G, Vorontsov E et al (2018) Learning normalized inputs for iterative estimation in medical image segmentation. Med Image Anal 44:1–13. 10.1016/j.media.2017.11.005 29169029 10.1016/j.media.2017.11.005

[22] Zeng Y, Tsui PH, Wu W, Zhou Z, Wu S (2021) Fetal ultrasound image segmentation for automatic head circumference biometry using deeply supervised attention-gated V-Net. J Digit Imaging 34:134–148. 10.1007/s10278-020-00410-5 33483862 10.1007/s10278-020-00410-5 PMC7887128

[23] Jia G, Lam HK, Xu Y (2021) Classification of COVID-19 chest X-Ray and CT images using a type of dynamic CNN modification method. Comput Biol Med 134:104425. 10.1016/j.compbiomed.2021.104425 33971427 10.1016/j.compbiomed.2021.104425 PMC8081579

[24] Zhao C, Shi S, He Z et al (2023) Spatial-temporal V-Net for automatic segmentation and quantification of right ventricle on gated myocardial perfusion SPECT images. Med Phys 50:7415–7426. 10.1002/mp.16805 37860998 10.1002/mp.16805

[25] Huang G, Liu Z, Pleiss G, Maaten LV, Weinberger KQ (2022) Convolutional networks with dense connectivity. IEEE Trans Pattern Anal Mach Intell 44:8704–8716. 10.1109/tpami.2019.2918284 31135351 10.1109/TPAMI.2019.2918284

[26] Heo J, Lim JH, Lee HR et al (2022) Deep learning model for tongue cancer diagnosis using endoscopic images. Sci Rep 12:6281. 10.1038/s41598-022-10287-9 35428854 10.1038/s41598-022-10287-9 PMC9012779

[27] Andaloro C, Kwon KW, Park SH et al (2024) Deep learning algorithm for the automated detection and classification of nasal cavity mass in nasal endoscopic images. PLoS One 19:e0297536. 10.1371/journal.pone.0297536 38478548 10.1371/journal.pone.0297536 PMC10936791

[28] Chen Y, Wang L, Dong X et al (2023) Deep learning radiomics of preoperative breast MRI for prediction of axillary lymph node metastasis in breast cancer. J Digit Imaging 36:1323–1331. 10.1007/s10278-023-00818-9 36973631 10.1007/s10278-023-00818-9 PMC10042410

[29] Guo S, Wang L, Chen Q et al (2022) Multimodal MRI image decision fusion-based network for glioma classification. Front Oncol 12:819673. 10.3389/fonc.2022.819673 35280828 10.3389/fonc.2022.819673 PMC8907622

[30] Yan CH, Tong CCL, Penta M et al (2018) Imaging predictors for malignant transformation of inverted papilloma. Laryngoscope 129:777–782. 10.1002/lary.27582 30515841 10.1002/lary.27582

