# Supplementary material for: Deep learning using nasal endoscopy and T2-weighted MRI for prediction of sinonasal inverted papilloma-associated squamous cell carcinoma: an exploratory study
Source: Eur Radiol Exp. 2025 Jul 21;9:68. doi: 10.1186/s41747-025-00610-0 (PMC12279620; doi:10.1186/s41747-025-00610-0)
Supplement: Supplementary file 1 — ELECTRONIC SUPPLEMENTARY MATERIAL [file 41747_2025_610_MOESM1_ESM.pdf]

Deep learning using nasal endoscopy and T2-weighted MRI for prediction of sinonasal inverted papilloma-associated squamous cell carcinoma: an exploratory study

ELECTRONIC SUPPLEMENTARY MATERIAL

Eur Radiol Exp (2025) Ren J, Ren Z, Zhang D, Yuan Y, Qi M.

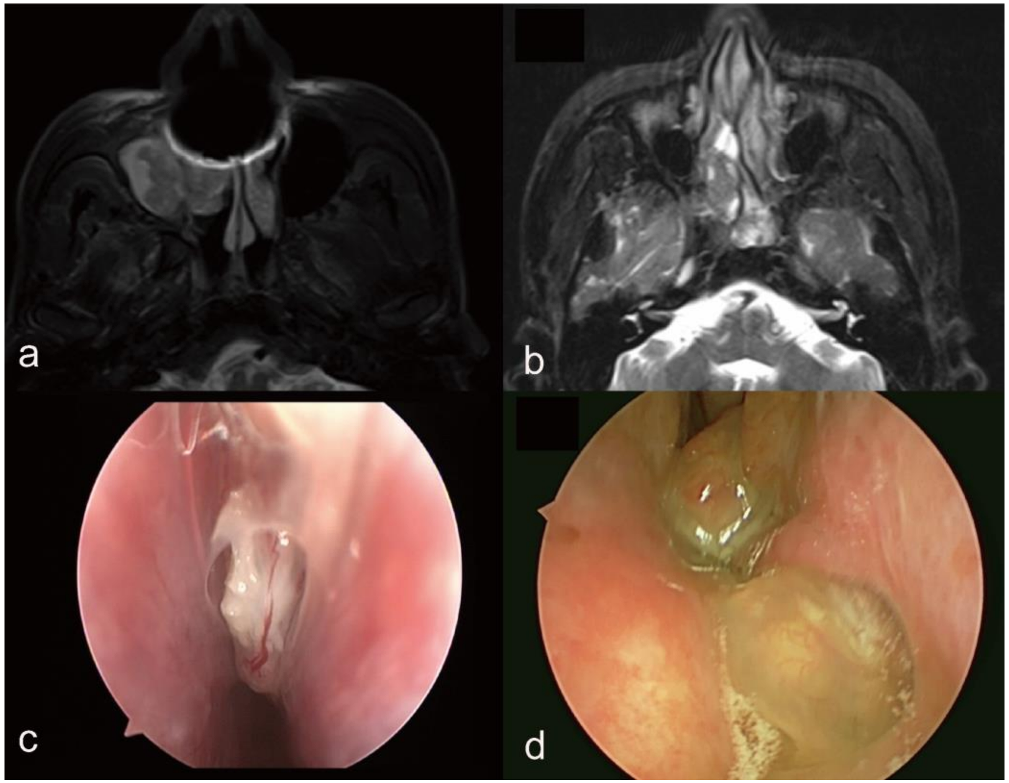

Supplementary Fig. 1 Representative cases featuring poor image quality. a. An MRI image marred by metal artifacts. b. An MRI image affected by motion artifacts. c. An endoscopic image where excessive mucus obscures the lesion. d. An endoscopic image under poor lighting conditions.
